# XRD, FTIR and ultrasonic investigations of cadmium lead bismuthate glasses

**DOI:** 10.1038/s41598-023-39489-5

**Published:** 2023-08-07

**Authors:** Amin Abd El-Moneim, M. A. Azooz, Hassan A. Hashem, A. M. Fayad, R. L. Elwan

**Affiliations:** 1 Physics Department, Faculty of Science, Zagazig University, Zagazig, 44519 Egypt; 2 Glass Research Department, National Research Centre, Dokki, Cairo, Egypt; 3 Nano Materials Research Lab., Physics Department, Faculty of Science, Zagazig University, Zagazig, 44519 Egypt

**Keywords:** Materials science, Physics

## Abstract

Cadmium lead bismuthate glasses in the system xCdO–(1−x)[0.5PbO + 0.5Bi_2_O_3_](40 mol% ≤ x ≤ 90 mol%) were successfully prepared by melt-quenching method. The structural and elastic properties have been investigated using XRD, FTIR and ultrasonic pulse–echo techniques. The XRD patterns confirmed the amorphous nature of the samples prepared. Density and ultrasonic velocity data were used to evaluate various elastic properties. Addition of CdO gave rise to decreased density and molar volume and increased elastic moduli, micro-hardness, and Debye temperature. The FTIR analysis revealed that increasing CdO content enhances the BiO_6_ octahedral sites at the expense of the BiO_3_ and PbO_4_ units. This results in the formation of Pb–O–Bi(6) and Bi(3)–O–Bi(6) linkages in the glass network, which stiffen the structure and improve the elastic properties. A correlation between elastic and compositional parameters was achieved on the basis of theories and approaches in the field.

## Introduction

Glasses containing heavy metal oxides Bi_2_O_3_ and PbO have high refractive index and low melting point. Lead containing glasses find a lot of applications in the field of solder glasses, table wares and optical lenses. Although Bi^3+^ and Pb^2+^ ions have the same 6s^2^ electronic configurations, in toxicity, bismuth is much safer than lead. Bi_2_O_3_ is suitable for forming glass with high refractive index, non-toxicity and wide transmission range^1^. For these reasons, Bi-based glasses can be substituted for the Pb-containing glasses. Now, bismuth-contained glass systems are applied in optical and electronic devices, mechanical sensors, and reflecting windows^2,3^.

The structural role of Bi_2_O_3_ in glasses is complicated because it is not a classical glass former. During recent years, there has been increasing interest in the synthesis, structure and physical properties of heavy metal oxide glasses containing Bi_2_O_3_ due to their high refractive index, high infrared transparency, increased third order nonlinear optical susceptibility. Due to the high polarizability and small field strength of Bi^3+^ ions, a glass network of BiO_3_ and BiO_6_ structural units may be built in the presence of SiO_2_ and B_2_O_3_^4^. PbO has dual role in the structure of glasses, one as a glass modifier (if Pb–O is ionic) and the other as glass former (if Pb–O is covalent)^5^. In a previous study^4,6^, it has been reported that Bi^3+^ ions can be present in the glass structure as three [BiO_3_]—or six [BiO_6_]-coordinated, existing separately or in the mixture. Meanwhile, Pb^2+^ ions can be present as three [PbO_3_]—or four [PbO_4_]—coordinated.

Cadmium is used as a barrier inside reactors to absorb neutrons and control nuclear fission^7^. FTIR studies of pure CdO nano-composites revealed an absorption peak around 420 cm^−1^, which is assigned to the Cd–O bond^8^. Due to their application in nonlinear optical materials, cadmium-doped glasses have attracted the attention of researchers during recent years^7^. Glasses from the binary CdO–B_2_O_3_ system (CdO = 50–90 mol %) were prepared by melting followed by annealing technique^9^. The FTIR spectra of these glasses indicated that CdO is consumed in the conversion of some BO_3_ groups to BO_4_ groups as many of alkali and alkaline earth oxides. At high CdO content, Cd^2+^ ions are assumed to behave as modifier besides forming CdO_4_ groups. Some Nd^3+^ doped cadmium borate glasses were prepared and characterized optically^10^. The absorption spectra have been analyzed on the basis of Judd–Ofelt theory. Effect of Na_2_O on structural and thermal properties of cadmium borate glasses has been investigated by Pavai et al.^11,12^. XRD and SEM results confirmed the amorphous nature of the glass samples, whereas the FTIR results indicated that the structural role played by Na_2_O and CdO ions preferentially get incorporated as modifier and former respectively. The influence of CdO and gamma irradiation on the IR absorption spectra of borosilicate glass was investigated^12^. The infrared results identified the presence of BO_3_, BO_4_, SiO_4_ structural units and B–O–Cd linkages in these glasses. Structure and properties of binary CdO–B_2_O_3_ and ternary xCdO·(50−x)MnO·50B_2_O_3_ glasses (0 ≤ x ≤ 50 mol%) were extensively studied^13^. The fraction of four coordinated boron atoms $\left( {N_{4} } \right)$ was found to be does not change with increasing CdO content. Cadmium-doped lead borate glasses are characterized with the help of IR and Raman spectroscopy^14^. The conversion of three-fold to four-fold coordination of boron atoms was confirmed. On the other hand, the absence of the IR absorption band at 840/cm suggests that the CdO_4_ structural units do not formed in these glasses.

Elastic properties are suitable in describing the glass structure as a function of its composition and network connectivity. These properties help in assessing the mechanical strength and structure of glasses. The literature survey indicates that reports on elastic properties of glasses containing CdO are limited. In the current work, for the first time, the structural and elastic properties of cadmium lead bismuthate glasses in the system xCdO–(1−x)[0.5PbO + 0.5Bi_2_O_3_] (40 mol% ≤ x ≤ 90 mol%) have been investigated using XRD, FTIR and ultrasonic pulse-echo techniques. The basic building units in the glass network are identified and the structural changes induced by CdO addition were established. A study on the correlation between elastic and compositional parameters has also been carried out to obtain information regarding the local structure of these glasses.

## Experimental

### Sample preparation

**Figure 1:**
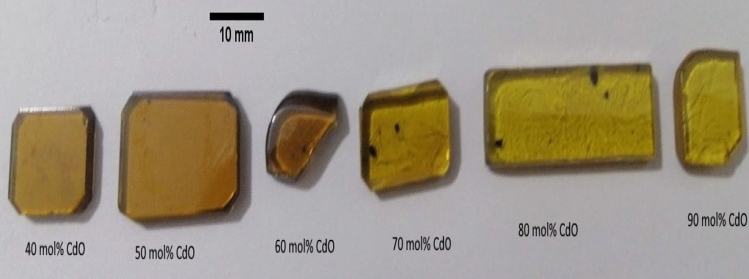
Photograph of the prepared xCdO–(1−x)[0.5PbO−0.5Bi_2_O_3_] glass samples.

The investigated glass samples in the present study were prepared from laboratory chemicals with purity > 99%, including cadmium carbonate CdCO_3_ (Fluka, Germany) for CdO, lead tetroxide Pb_3_O_4_ (TECHNO FARMACHEM, India) for PbO and Bi_2_O_3_ (Fluka, Germany) was added as such. The required amount of different chemicals for producing 100 g glass melt was weighed using a single pan balance having an accuracy of ± 0.001 g. The chemical composition and code of the prepared samples are listed in Table 1. The mixture is put in a porcelain crucible and heated inside a SiC-heated furnace (Vecstar, UK) to 1050 °C for 2 h for melting. The melts were rotated at intervals of 20 min to arrive complete mixing and acceptable homogeneity. The homogeneous molten mixtures were casted into a warmed stainless-steel mold to get the required dimensions. Then the prepared samples were immediately transferred to an annealing muffle furnace regulated at 320 °C—less than the glass transition temperature—to avoid the mechanical strain developed during the quenching process. The muffle was switched off after 1 h and left to cool with the glass inside to room temperature at a rate of 30 °C/h. Finally, the prepared glass samples were polished and their surfaces were made perfectly parallel and smoothened by diamond disc and diamond powder. The samples prepared were non-hygroscopic and chemically stable. Figure 1 shows the photograph of glass samples prepared in the present work.

**Table 1:** Chemical compositions (in mol%) and codes of the prepared glass samples.

Sample code	CdO	PbO	Bi_2_O_3_
Cd40	40	30	30
Cd50	50	25	25
Cd60	60	20	20
Cd70	70	15	15
Cd80	80	10	10
Cd90	90	5	5

### X-ray diffraction (XRD) analysis

The preparedglass samples were subjected to powder X-ray diffraction (XRD), using Bruker AXA diffractometer (Germany) with graphite monochromatized Cu–Kα radiation operating at 40 kV and 10 mA and scanning rate 10°/min was used for x-ray measurements. The measurements were carried out in the range 5° ≤ $2\theta$ ≥ 80°.

### Fourier transform infrared (FTIR) absorption measurements

FTIR technique is one of the spectroscopic techniques used to identify the basic building units in the glass network. FTIR absorption spectra of glasses prepared were recorded at room temperature in the wave number range from 400 to 1500/cm by a Fourier transform computerized IR spectrometer type FTIR 4600 JASCO Corp (Japan) using the KBr disc technique. The glasses were examined in the form of pulverized powder which was mixed with KBr with the ratio 1:100 mg glass powder to KBr, respectively. The weighed mixtures were then subjected to a pressure of 5 tons/cm^2^ to produce clear homogeneous discs.

### Density measurement and molar volume calculation

The densities of the samples that were prepared were determined at room temperature using an analytical balance A&D company limited, model GR-200, with a repeatability of 0.1 mg, and a density determination kit based on the standard test method ASTM-D792-13. The Archimedes technique was used, with distilled water as the buoyant liquid, and the following formula was applied:

1 $$\rho = \frac{{W_{a} \,\rho_{w} }}{{\left( {W_{a} - W_{w} } \right)}}$$

where ρ_w_ represents the density of distilled water at room temperature, while W_a_ and W_w_ represent the weights of the sample in air and distilled water, respectively. In order to assess the uncertainty associated with the density (ρ) and molar volume (Vm), three measurements were taken for each sample. The formula used to determine the uncertainty in densities and molar volume is based on the standard deviation (σ) and the number of independent observations (n). The standard uncertainty can be calculated using the following expression^15^: $u = \frac{\sigma }{\sqrt n }$. Empirical densities (crystalline densities) were calculated using equation $\rho_{C} = \sum\limits_{i} {x_{i} \,\rho_{i} }$, where $\rho_{i}$ is the density of *i*th oxide component in the crystalline phase and $x_{i}$ is its molar fraction. The molar volume deals directly with the spatial structure of the glass network. The molar volume of each glass sample was calculated with the help of the following equation:

2 $$V_{M} = \frac{1}{\rho }\sum\limits_{i} {x_{i} \,M_{i} }$$

where $M_{i}$ is the ith component molecular weight. The volume occupied by crystalline phases in the glass network is known as empirical (crystalline) molar volume. Assuming the mixture of oxide components to be an ideal solution we also calculated the empirical molar volume for each glass composition using the relation $V_{C} = \sum\limits_{i} {x_{i} \,M_{i} /\rho_{i} }$.

### Ultrasonic measurements

The ultrasonic pulse-echo technique is a non-damaging technique that capable of determining elastic properties of glasses. The measurements of longitudinal and shear ultrasonic wave velocities in the prepared glass samples were made at room temperature and at 4 MHz frequency. The ultrasonic pulse travels through the sample bonded to the transducer and the echo is registered each time it returns to the transducer. The flaw detector (KRAUTKRAMER USM 36) was applied to measure the time interval between the pulse and its echo (or two successive echoes) ($\Delta \,t$). Ultrasonic velocities were determined by dividing the thickness of the sample by $\Delta \,t$.

Elastic properties are informative because they are related to the structure of the glass network. The ultrasonic velocities and density data allow the determination of various elastic properties using the standard relations; longitudinal modulus $L = \rho \,V_{\ell }^{{^{2} }}$, shear modulus $S = \rho \,V_{s}^{{^{2} }}$, bulk modulus $K = \rho \,\left( {V_{\ell }^{2} - \frac{4}{3}\,V_{s}^{2} } \right)$, Poisson’s ratio $\mu = \frac{(L - 2\,S)}{{2(L - S)}}$, Young’s modulus $E = 2\,S(1 + \mu_{\exp } )$, Micro-hardness $H = \frac{S}{3}(1 - 2\mu_{\exp } )$ and Debye temperature $\theta_{D} = \frac{h}{B}\left[ {\frac{{3\,\,N_{a} \,}}{{4\,\pi \,\,\overline{V}\,}}} \right]^{\frac{1}{3}} V_{mean}$. In these relations, $V_{l}^{{}}$ and $V_{s}$ are the respective longitudinal and shear ultrasonic velocities, $N_{a}$ is Avogadro’s number, $B$ is Boltzmann’s constant, $h$ is Planck’s constant and $V_{{mean}} = \left[ {\frac{1}{3}\left( {\frac{1}{{V_{\ell }^{3} }}{\text{ + }}\frac{{\text{2}}}{{{\text{V}}_{{\text{s}}}^{{\text{3}}} }}} \right)} \right]^{{^{ - } \frac{1}{3}}}$ is the mean ultrasonic velocity.

## Results and discussion

### X-ray diffraction (XRD) analysis

**Figure 2:**
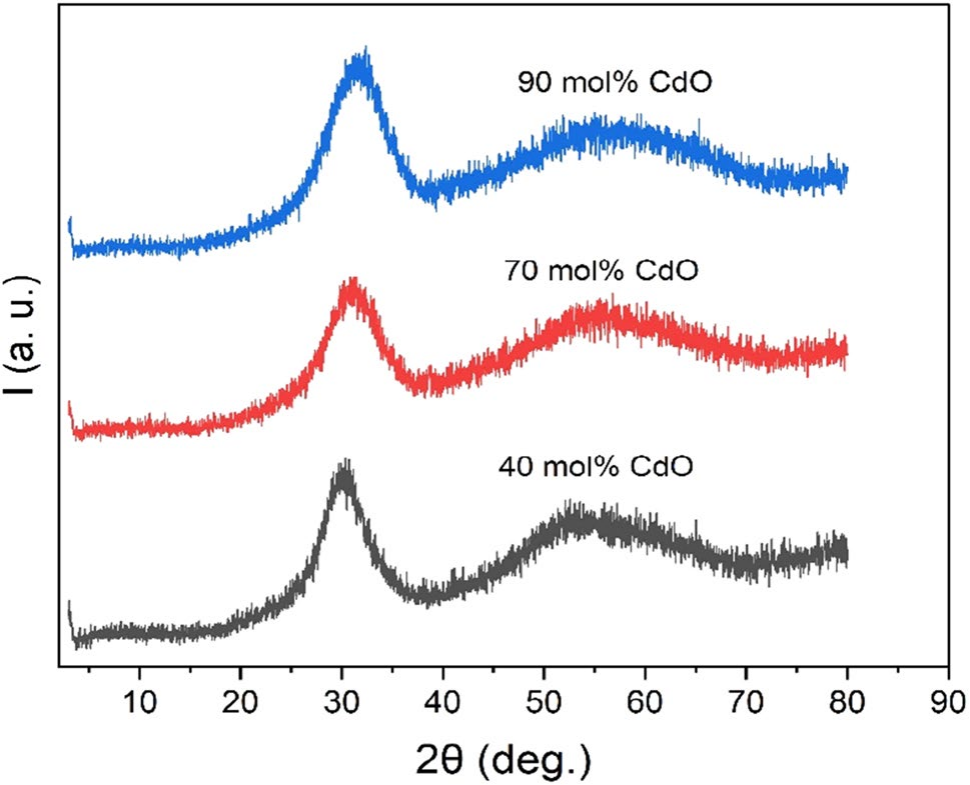
The X-ray diffraction (XRD) patterns of Cd40, Cd70 and Cd90 glass samples.

X-ray diffraction is one of the simplest techniques, which was used by researchers to establish the amorphous nature of oxide glasses^16^. Figure 2 depicts the X-ray diffraction patterns for Cd40, Cd70 and Cd90 glass samples. The absence of sharp Bragg peaks and presence of broad humps confirms the amorphous nature of these samples ^16^. The samples lack in long-range atomic periodicity in their networks.

### FTIR spectral analysis

**Figure 3:**
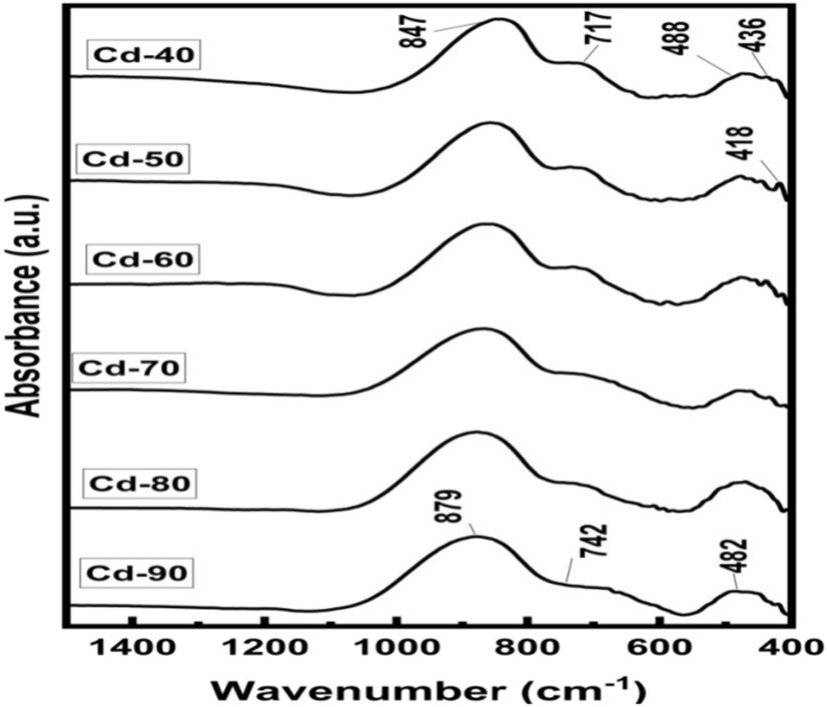
Measured FTIR absorption spectra of xCdO–(1−x)[0.5PbO + 0.5Bi_2_O_3_] glasses.

IR spectroscopy studies were used to get essential information about the arrangement of the structural units of the glass samples. Figure 3 shows the measured FTIR absorption spectra of xCdO–(1−x)[0.5PbO−0.5Bi_2_O_3_] glass samples under investigation. These spectra are dominated by two broad absorption bands in the range 400–550/cm and 750–1050/cm and a small but well-distinguished band centered at 717/cm. The broad band extending from 400 to 550/cm is attributed to bending vibration of Bi–O bonds in BiO_6_ octahedral units and symmetric bending vibration of Pb–O bonds in PbO_4_ tetragonal pyramids^17,18^. The presence of this band confirms the former role of PbO and Bi_2_O_3_ in the matrix of the studied glasses. Some author’s attributed the FTIR band at 720/cm in the ZnO modified bismuth silicate glasses^19^ and that at 715/cm in ternary Bi_2_O_3_–B_2_O_3_–CuO glasses^20^ to the symmetric stretching vibrations of Bi–O bond in BiO_3_ structural units. The appearance of this band in investigated glasses at 717/cm (Fig. 3) evidences the existence Bi^3+^ ions not only as BiO_6_ units but also as BiO_3_ units. The broad band in the wavenumber range 750–1050/cm is assigned to the vibrations of Bi–O–Bilinkages^19^. With increasing CdO content, the intensity of this band has been observed to increase whereas that of BiO_3_ units decreases. This suggests that the gradual increase in the concentration of cadmium oxide in xCdO–(1−x)[0.5PbO−0.5Bi_2_O_3_] glasses enhances the BiO_6_ octahedral sites in the glass matrix at the expense of BiO_3_ groups. The low frequency peak at 418/cm, which was observed in all samples is may be attributed to vibrations of Cd^2+^ metal cations.

**Figure 4:**
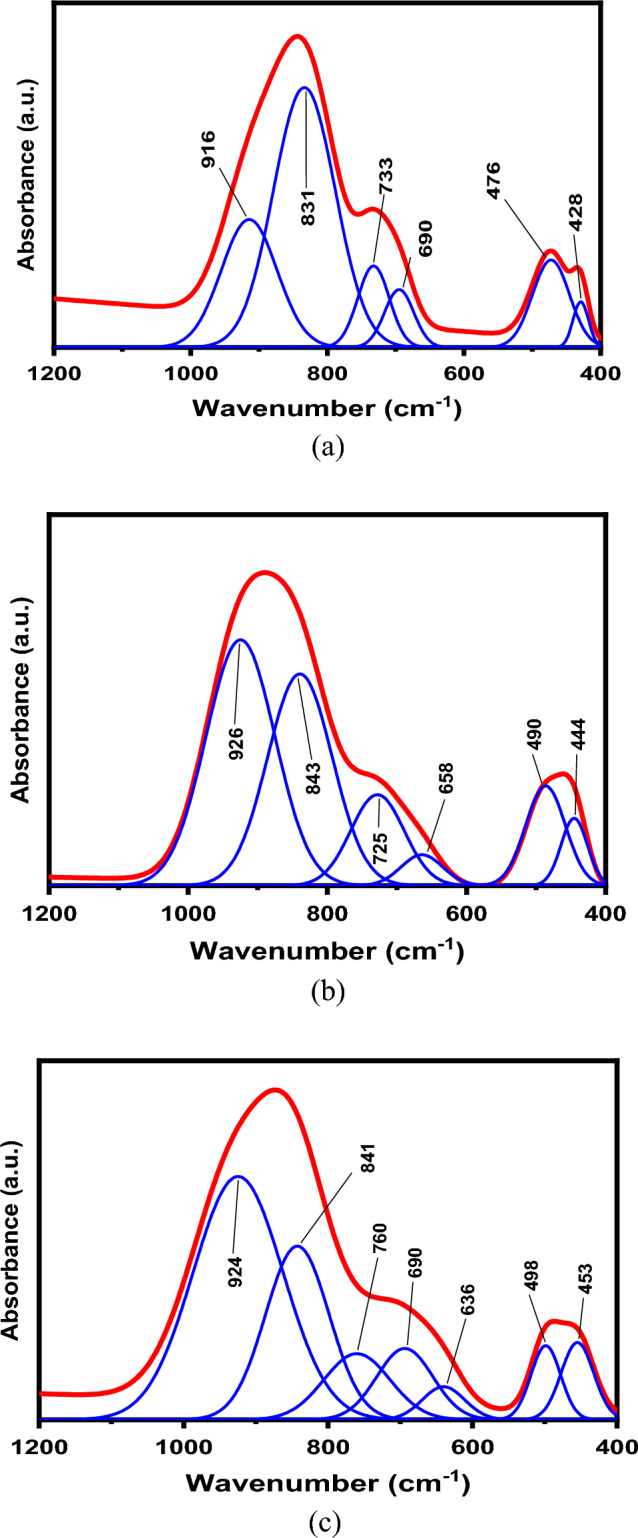
Fitted FTIR absorption spectra for (**a**) Cd40 glass sample, (**b**) Cd80 glass sample and (**c**) Cd90 glass sample.

Because the observed bands are very broad and asymmetric, the deconvolution process should be applied to the measured FTIR spectra to decompose each broad absorption band to deconvoluted peaks (component bands). Each peak has two characteristic parameters, which are the center (C) and the relative area (A). The center is related to some type of vibrations of a specific structural group, whereas the relative area (A) is proportional to the concentration of this structural group. Figure 4 displays the deconvoluted peaks for Cd40, Cd80 and Cd90 glass samples. The Gaussian distribution was applied in the present deconvolution. The peak centers and their corresponding assignments are shown in Table 2.

**Table 2:** FTIR absorption peaks in xCdO–(1−x)[0.5PbO + 0.5Bi_2_O_3_] glasses and their assignments.

Peak center ± 2 (/cm)	Associated vibrational mode	Refs.
428–453	Bi–O bending vibration in BiO_6_ octahedral units	^17^
476–498	symmetric bending vibration of Pb–O in PbO_4_ tetragonal pyramid	^21,22^
636	Various modes of Bi–O vibration in BiO_6_ units and/or Cd–O in CdO	^23,24^
	Bi–O^−^ stretching vibrations in BiO_6_ units	^17^
690–658	Pb–O symmetrical bending vibrations	^21^
760–725	Pb–O bond vibrations of PbO_n_ units with n = 3 or 4	^21,25^
831–843	Symmetric stretching vibrations of Bi–O bonds in BiO_3_ units	^17,25^
916–926	Symmetric stretching vibrations of Bi–O bonds in BiO_6_ units or Pb–O–Bi, Pb–O–Cd, Bi–O–Cd, or Bi(3)–O–Bi(6) specific vibrations	^25^

The general characteristics of the fitted FTIR absorption spectra for the investigated xCdO–(1−x)[0.5PbO + 0.5Bi_2_O_3_] glasses can be summarized as follows;

1. The broad band extending from 400 to 550/cm was deconvoluted to two peaks at about 428 and 476/cm, which are related to BiO_6_ and PbO_4_ units, respectively. The intensity of these peak has been observed to increase with increasing CdO.
2. The broad band extending from 550 to 1100/cm was deconvoluted to four peaks at about 690, 733, 831 and 916/cm. An additional absorption peak centered at 636/cm was observed only in Cd90 sample. This may be attributed to the metal–oxygen starching of CdO. The peak centered at 620/cm has been assigned to Cd–O bonds^23^.

**Figure 5:**
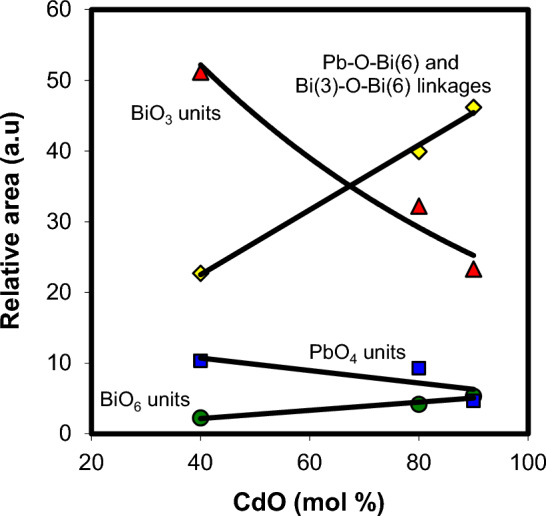
Composition dependence of the relative area of BiO_3_, BiO_6_, and PbO_4_ structural units and Pb–O–Bi and Bi–O–Bi linkages.

The most important condition for the formation of BiO_3_ structural units in the glass network is the appearance of the 830/cm band in the FTIR spectra^17^. In the present study, this band appears in the wave number range 831–843/cm, which proofs the coexistence of BiO_6_ and BiO_3_ units in the structure of xCdO–(1−x)[0.5PbO + 0.5Bi_2_O_3_] glasses. Figure 5 shows that the relative area of BiO_3_- and PbO_4_—relating absorption bands decrease whereas that of bands characterizing BiO_6_ units and Pb–O–Bi(6), and Bi(3)–O–Bi(6) linkages increase with the addition CdO. This may be explained by accepting the assumption that the addition of the cadmium oxide declines the formation of BiO_3_ and PbO_4_ units in favor of the formation of BiO_6_ units in the glass. The presence of extra BiO_6_ units and formation of Pb–O–Bi(6), and Bi(3)–O–Bi(6) linkages is expected to increase the compactness of the glass structure with increasing CdO content. This reflected the glass modifier role of CdO, which results in the change in the coordination number of bismuth ions from three to six. The structure of the studied glasses is assumed to be built up by BiO_3_, BiO_6_, and PbO_4_ structural units. These structural units are connected to each other through Pb–O–Bi, and Bi–O–Bi linkages.

### Density and molar volume

**Figure 6:**
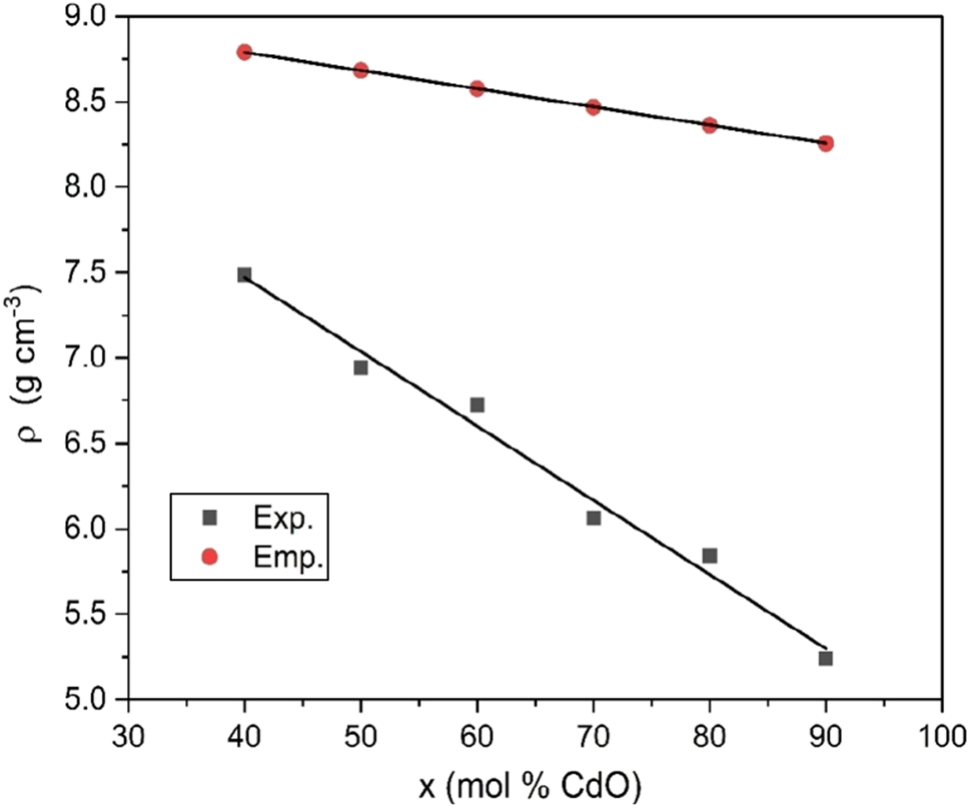
Composition dependence of density in xCdO–(1−x)[0.5PbO + 0.5Bi_2_O_3_] glasses.

The density is one of the simplest tools used to detect changes in the glass structure^26–29^. Also, the molar volume can be preferably used to describe the network structure and the arrangement of the building units in glasses^26–29^. Table 3 summarized the physical properties of xCdO–(1−x)[0.5PbO + 0.5Bi_2_O_3_] glasses under investigation. The variation of measured and empirical densities with the molar fraction of CdO is shown in Fig. 6. As clearly observed, the measured density values decrease monotonically from 7.4888 to 5.2401 g/cm^3^ with increasing the molar ratio of CdO from 40 to 90 mol%. This behavior is not only attributed to the lighter molar mass of CdO (128.41 g/mol) than that of Bi_2_O_3_ (465.96 g/mol) and PbO (223.20 g/mol) but also attributed to the relative changes in the density values between CdO (8.15 g/cm^3^), Bi_2_O_3_ (8.9 g/cm^3^) and PbO (9.53 g/cm^3^). The empirical density values also decreased, but with a slower rate, over the entire composition range studied. As a result $(\rho - \rho_{C} )$ value increases with increasing CdO content, which supports the amorphous nature of samples as confirmed from the XRD results in section “X-ray diffraction (XRD) analysis”.

**Table 3:** Physical and compositional properties of xCdO–(1−x)[0.5PbO + 0.5Bi_2_O_3_] glasses.

Sample code	$\rho$ ± 0.0002 (g/cm^3^)	$V_{M}$ ± 0.01 (cm^3^/mol)	$C_{t}$	$G_{t}$ (kcal/cm^3^)
Cd40	7.4888	34.47	0.499	7.259
Cd50	6.9419	34.07	0.503	7.373
Cd60	6.7270	31.94	0.506	7.486
Cd70	6.0655	31.86	0.510	7.600
Cd80	5.8427	29.38	0.513	7.713
Cd90	5.2401	28.63	0.517	7.827

**Figure 7:**
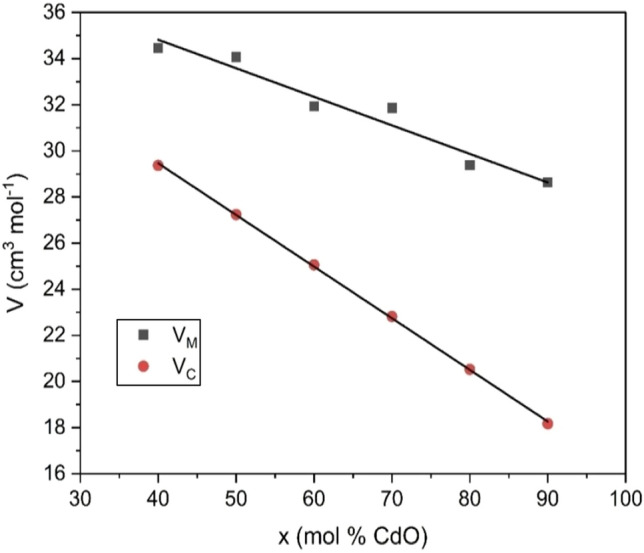
Variation of molar volume with mol% of CdO in xCdO–(1−x)[0.5PbO + 0.5Bi_2_O_3_] glasses.

In the present investigation, the molar volume of xCdO–(1−x)[0.5PbO + 0.5Bi_2_O_3_] glasses decreased monotonically from 34.47 to 28.63 cm^3^/mol with increasing CdO content from 40 to 90 mol% (Fig. 7). The empirical molar volume $V_{C}$ behaves the same trend but with a higher rate of decrease. This indicates structure compactness with CdO addition. This parallel behavior of density and molar volume with composition was reported in the literature for numerous glasses^30–33^. Change in the molar volume might be ascribed to the lattice rearrangements caused by the substitution of PbO and Bi_2_O_3_ by CdO resulting in decreasing the ring size, leading to the structural closure. Thus, the decrease in molar volume with increasing CdO content can be explained in two ways as follows:

1. The atomic radii of ingredient elements were changed in the order Cd (1.52 nm) < Pb (1.63 nm) < Bi (1.81 nm). Moreover, the ionic radius values were 0.119 nm for Pb^2+^ ion and 0.103 nm for Cd^2+^ and Bi^3+^ ions. Thus, the substitution of Pb^2+^ and Bi^3+^ ions by Cd^2+^ ion is expected to contracts the structure and decreased the molar volume.
2. Based on FTIR data obtained, the addition of CdO declines the presence of the structural units BiO_3_ and PbO_4_ in favor of the presence of BiO_6_ structural units in the glass network. This results in the formation of Bi(3)–O–Bi(6) and Pb–O–Bi(6) linkages at the expense of Pb–O–Bi(3) linkages. The curves of the radial distribution function (RDF) obtained by the Fourier transformation for PbO–B_2_O_3_ glasses concluded that the well-separated peaks due to Pb–O and Pb–Pb correlations appear at about 0.23–0.25 and 0.40 nm, respectively^34^. Also, X-ray diffraction studies of binary Bi_2_O_3_–B_2_O_3_ glasses revealed two groups of Bi–O distances at about 0.19 and 0.25 nm^35^. In the light of these data, the formation of Bi(3)–O–Bi(6) and Pb–O–Bi(6) linkages at the expense of Pb–O–Bi(3) linkages in the present xCdO–(1−x)[0.5PbO + 0.5Bi_2_O_3_] glasses is expected to increase the compactness of their structure and decrease the molar volume.

The increase in the difference between glass and crystalline molar volumes with increasing CdO content in the glass confirms the amorphous nature of samples.

### Ultrasonic velocity and elastic properties

**Figure 8:**
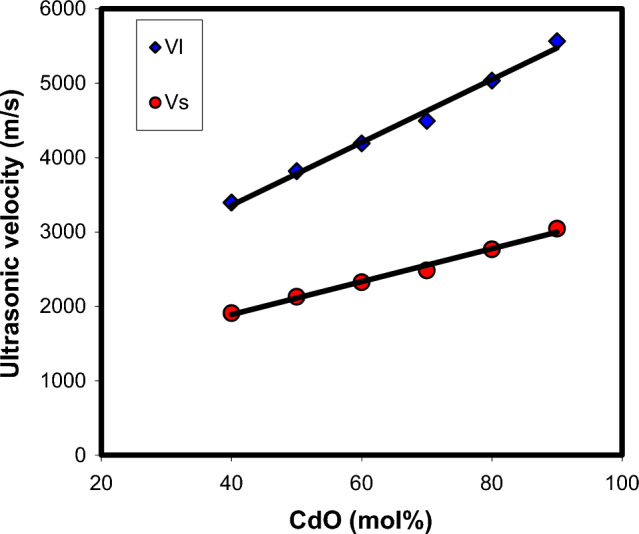
Composition dependence of ultrasonic velocities in xCdO–(1−x)[0.5PbO + 0.5Bi_2_O_3_] glasses.

**Figure 9:**
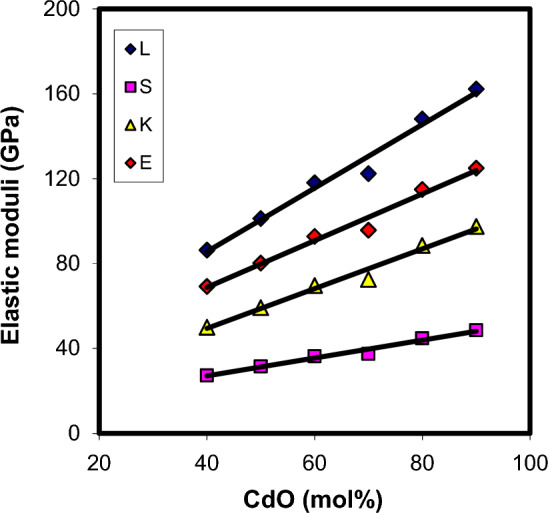
Composition dependence of elastic moduli in xCdO–(1−x)[0.5PbO + 0.5Bi_2_O_3_] glasses.

**Figure 10:**
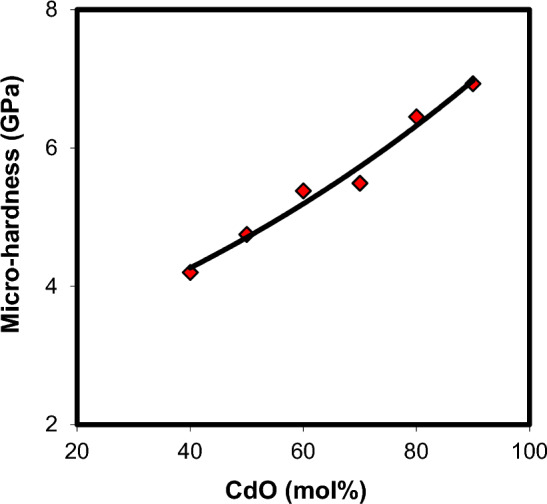
Composition dependence of micro-hardness in xCdO–(1−x)[0.5PbO + 0.5Bi_2_O_3_] glasses.

**Figure 11:**
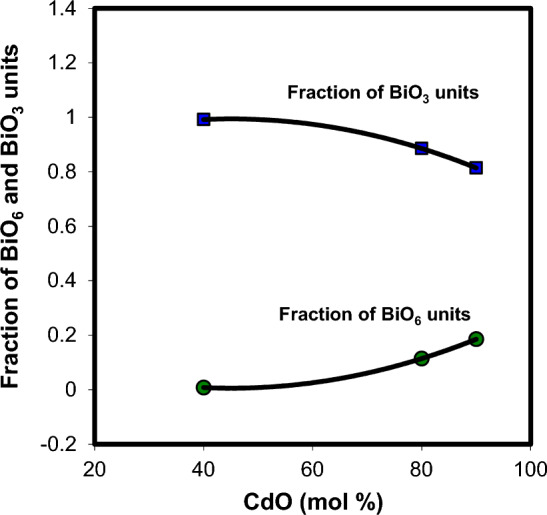
Composition dependence of fraction of BiO_6_ and BiO_3_units inxCdO–(1−x)[0.5PbO + 0.5Bi_2_O_3_] glasses.

Changes in the glass structure due to addition of a network modifier and/or a network former can be directly reflected in ultrasonic velocities and elastic properties. Thus, the composition dependence of ultrasonic velocities and elastic moduli may yield information about the dimensionality and nature of bonding in the glass. The results of longitudinal and shear ultrasonic velocities measurements, along with the determined values of elastic moduli, micro-hardness, Debye temperature and Poison's ratio are listed in Table 4 for xCdO–(1−x)[0.5PbO + 0.5Bi_2_O_3_] glasses studied. One can observe from Fig. 8 that both longitudinal and shear velocities increase linearly with the substitution of (0.5PbO + 0.5Bi_2_O_3_) by CdO. Also, the elastic moduli (Fig. 9) and micro-hardness (Fig. 10) are improved with the progressive addition of CdO. Generally, elastic moduli of the glass are strongly dependent on the concentration of basic structural units of the constituent oxides and types of bonds between these units. The FTIR results obtained indicated that the increase in the concentration of cadmium oxide enhances the BiO_6_ octahedral sites in the glass matrix at the expense of BiO_3_and PbO_4_ groups. As shown in Fig. 5, the relative areas of absorption band characterizing BiO_3_ and PbO_4_ units decrease, whereas that of BiO_6_ units increases. The fraction of the six coordinated bismuth atoms (or fraction of BiO_6_ units) was calculated from the relation $N_{6} = A_{6} /\,(A_{3} + A_{6} )$, where $A_{6}$ is the area under IR peaks characterizing BiO_6_ and $A_{3}$ is that under IR peaks characterizing BiO_3_ units. The $N_{6}$ values changed from 0.0081 in Cd40 sample to 0.1855 in Cd90 sample as shown Fig. 11. This behavior is quite similar to those of ultrasonic velocities and elastic moduli. It is well known that bulk modulus is a function of the cross-link density (coordination number of a network former cation minus 2) and number of network bonds per unit volume of the glass^36^. However, the BiO_6_, PbO_4_ and BiO_3_ structural units have a cross-link density of 4, 2 and 1, respectively. Thus, the presence of extra covalent bonds due to the formation of Pb–O–Bi(6) and Bi(3)–O–Bi(6) linkages at the expense of Pb–O–Bi(3) linkages is expected to increase the average cross-link density and number of network bonds per unit volume. This increases the rigidity and resists the deformation of glassy structure with increasing CdO content as evidenced by the increase in ultrasonic velocity, elastic moduli and micro-hardness.

**Table 4:** Ultrasonic velocities and elastic properties of xCdO–(1−x)[0.5PbO + 0.5Bi_2_O_3_] glasses.

Sample code	$V_{\ell }$ ± 20 (m/s)	$V_{s}$ ± 20 (m/s)	$L$ (GPa)	$S$ (GPa)	$E$ (GPa)	$K$ (GPa)	$\mu$	$H$ (GPa)	$\theta_{D}$ (K)	$d$
Cd40	3396	1909	86.38	27.28	69.24	50.01	0.269	4.20	234	2.18
Cd50	3819	2130	101.25	31.50	80.27	59.25	0.274	4.75	257	2.12
Cd60	4190	2324	118.09	36.34	92.87	69.63	0.278	5.38	282	2.09
Cd70	4493	2483	122.42	37.40	95.74	72.56	0.280	5.49	296	2.06
Cd80	5036	2768	148.19	44.76	114.91	88.51	0.284	6.45	332	2.02
Cd90	5565	3045	162.29	48.58	124.99	97.51	0.286	6.93	360	1.99

Poisson's ratio plays a dominant role in exploring the degree of connectivity and cross-link density of the glass structure. It is reported that, if the solid material has Poisson’s ratio < 0.3, then it has been counted in high cross-linking density materials^37^. Otherwise, it is considered in low cross-linking density materials. Three-dimensional glasses (high cross-link density materials) have Poisson’s ratio values extending from 0.1 to 0.2, meanwhile, two-dimensional glasses have Poisson’s ratio values extending from 0.3 to 0.5^37^. It can be seen from Table 3 that xCdO–(1−x)[0.5PbO + 0.5Bi_2_O_3_] glasses have Poisson’s ratio values < 3.0, which suggest that these glasses have high cross-linking density. These cross-links generate covalent bonds and resisting the transverse deformation. This evidenced the presence of PbO_4_, BiO_3_ and BiO_6_structural units within the matrix of these glasses.

**Figure 12:**
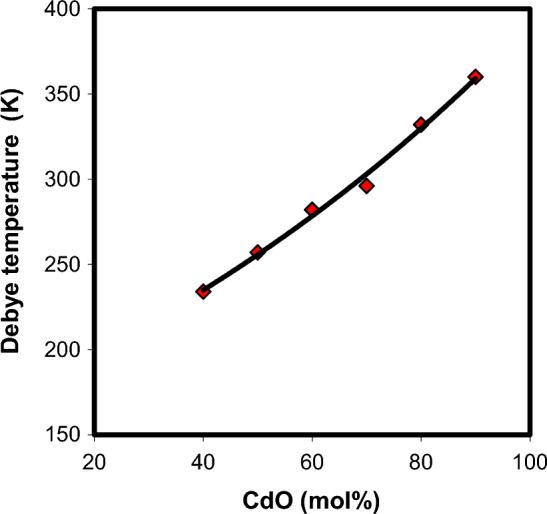
Variation of Debye temperature with mol% of CdO in xCdO–(1−x)[0.5PbO + 0.5Bi_2_O_3_] glasses.

Debye temperature is an important thermal parameter in the determination of elastic properties and atomic vibrations of solids. The increasing trend of Debye temperature in xCdO–(1−x)[0.5PbO + 0.5Bi_2_O_3_] glasses (Fig. 12) implies an enhancement in the compactness and rigidity with the substitution of (0.5PbO + 0.5Bi_2_O_3_) by CdO. This behavior is in consistent with the results of molar volume, ultrasonic velocities, elastic moduli, and micro-hardness.

**Figure 13:**
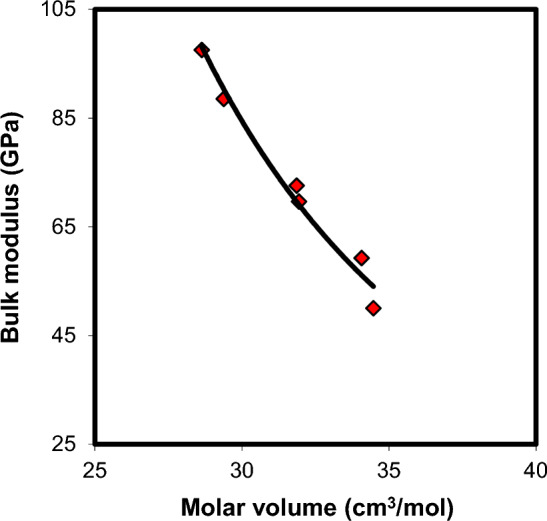
Bulk modulus-molar volume correlation in xCdO–(1−x)[0.5PbO + 0.5Bi_2_O_3_]glasses.

Gopal et al.^38^ and Rajendran et al.^39^ suggested bulk modulus—molar volume correlation according to the equation $K\,V_{M}^{b} = C$. In this relation $b$ and $C$ are two constants, their values are determined by the nature of bonding, and co-ordination polyhedral that are present in the glass structure. As shown in Fig. 13, the bulk modulus-molar volume relationship revealed an inverse proportionality for xCdO–(1−x)[0.5PbO + 0.5Bi_2_O_3_] glasses. The equation of the fitted curve is given by $K\,V_{M}^{3.21} = 5 \times 10^{6}$, with $b = \,3.21$, $C\, = \,5\, \times \,10^{6}$ and R^2^ = 0.963. A good bulk modulus-molar volume correlation was achieved in alkaline earth aluminoborate RO–Al_2_O_3_–B_2_O_3_ (R = Mg, Ca, Sr) glasses ($b = \,1.92$ and $C\, = \,3.7\, \times \,\left. {10^{4} } \right)$^40^, TiO_2_-doped borate CaO–Al_2_O_3_–B_2_O_3_ glasses ($b = \,4.38$ and $C\, = \,8.8\, \times \,1\left. {0^{7} } \right)$^41^, fluorotellurite BaF_2_–TeO_2_ glasses ($b = \,1.05$ and $C\, = \,1.0125\, \times \,\left. {10^{3} } \right)$^42^. This dependence of $b$ and $C$ values on the glass network supports Gopal et al.^38^ and Rajendran et al.^39^ approaches.

**Figure 14:**
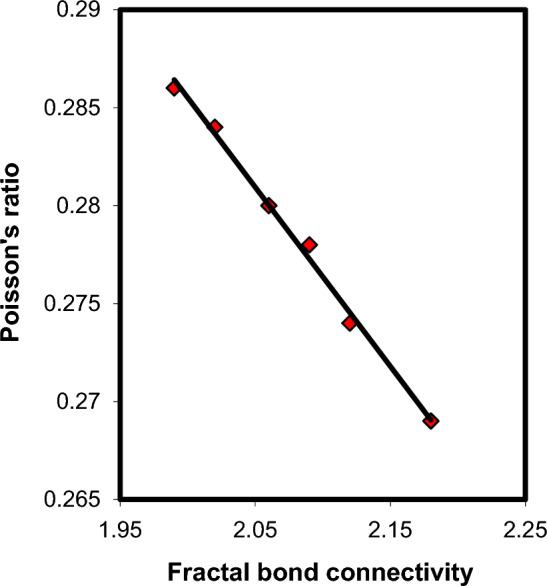
Poisson’s ratio-fractal bond connectivity correlation in xCdO–(1−x)[0.5PbO + 0.5Bi_2_O_3_] glasses.

Fractal bond connectivity ($d\, = \,4\,S\,/K$) can give information about the dimensionality of the glass structure^43^. This parameter testified the glass rigidity and mostly its values lie between 1 and 3. It has been found that, $d\, = \,1$ for 1D chain structure, $d\, = \,2$ for 2D layer network and $d\, = \,3$ for 3D network^43^. The $d\,$ values of the present xCdO–(1−x)[0.5PbO + 0.5Bi_2_O_3_] glasses lie between 1.99 and 2.18, which suggests 2D structures. One way to determine the elasticity of glasses is by studying Poisson’s ratio—fractal bond connectivity correlation on the basis of Abd El-Moneim’s approach^44^, according to the following equation:

3 $$\mu \, = A\, - \,\,Z\,d$$

where *A* and *z* are two constant, their values depend strongly on the glass network^44^. Figure 14 shows an inverse linear proportionality between $\mu \,$ and $\,d$, which confirms the validity of the semi-empirical Eq. (3) for the present xCdO–(1−x)[0.5PbO + 0.5Bi_2_O_3_] glasses. The equation of the fitted curve in this figure is given by $\mu \, = 0.468 - \,\,{0}.091\,d$, with R^2^ = 0.994. The present glasses have *A* and *z*values agree very well those reported previously for different glasses, like Li_2_O–V_2_O_5_–B_2_O_3_ glasses (*A* = 0*.*447 and *z* = 0*.*082)^45^, WO_3_–B_2_O_3_–MgO–TeO_2_ glasses (*A* = 0*.*466 and *z* = 0*.*09)^46^, Li_2_O–PbO–B_2_O_3_ glasses (*A* = 0*.*469 and z = 0*.*091)^47^ and PbO–CuO–B_2_O_3_ glasses (*A* = 0*.*473 and z = 0*.*094) glasses^48^. This supports the suitability of Abd El-Moneim’s approach^44^ for understanding the Poisson's ratio data of glasses.

**Figure 15:**
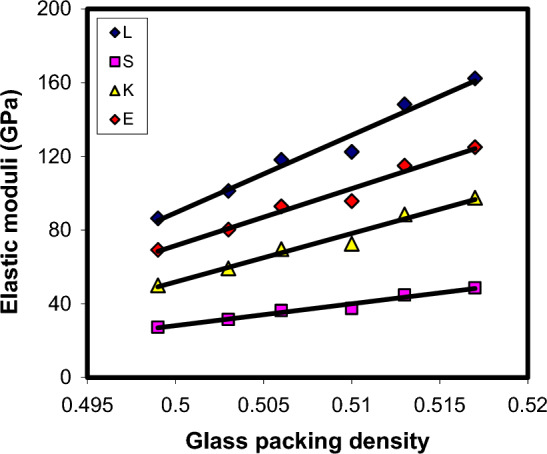
Elastic moduli—packing density correlation in xCdO–(1−x)[0.5PbO + 0.5Bi_2_O_3_] glasses.

**Figure 16:**
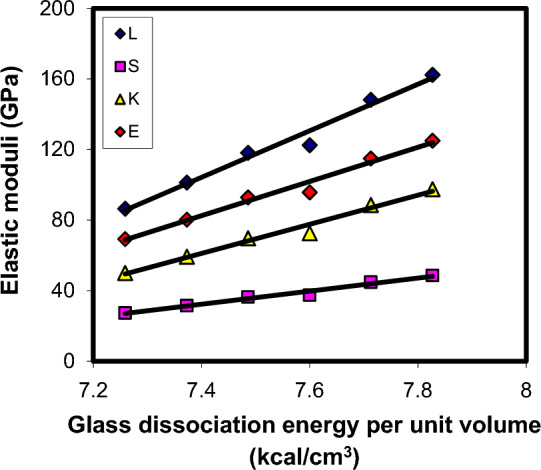
Elastic moduli—dissociation energy per unit volume in xCdO–(1−x)[0.5PbO + 0.5Bi_2_O_3_] glasses.

Understanding the obtained elastic moduli values of xCdO–(1−x)[0.5PbO + 0.5Bi_2_O_3_] glasses is based on the Makishima–Mackenzie’s theory^49,50^. It is a well-known fact that closely packed structures are rigid and have high values of packing density, whereas loosely packed structures are soft and have small values of packing density^49,50^. According to Makishima–Mackenzie's theory^49,50^, elastic moduli of glasses should show a forward relations with two compositional parameters, which are the total packing density ($C_{t} = \sum\limits_{i} {x_{i} } C_{i}$, where $C_{i}$ is the *i*th oxide component packing density) and total dissociation energy per unit volume ($G_{t} = \sum\limits_{i} {x_{i} } G_{i}$, where $G_{i}$ is the *i*th oxide component dissociation energy per unit volume). The calculated $C_{t}$ and $G_{t}$ values for xCdO–(1−x)[0.5PbO + 0.5Bi_2_O_3_] glasses are listed in Table 3. For CdO, PbO and Bi_2_O_3_ oxides, we have applied $C_{i}$ values of 0.5204, 0.3843 and 0.4985, respectively, whereas the applied $G_{i}$ values were 7.94, 6.05 and 7.56 kcal/cm^3^ respectively^51^. It was found that both $C_{t}$ and $G_{t}$ show an increase with the substitution of (0.5PbO + 0.5Bi_2_O_3_) mole by mole with CdO. These results agree well with the elastic moduli (Figs. 15 and 16), micro-hardness and Debye temperature results, which suggested an increase in the rigidity of the glass matrix with increasing CdO content. This confirms the applicability of Makishima–Mackenzie theory^49,50^ for predicting the elastic moduli of the investigated glasses.

The following equation, which correlating bulk modulus with the total packing density and Young’s modulus of ionic solid, was reported^49,50^:

4 $$K\, = \left[ {(m - n)\,m/24\pi } \right]\,\beta \,\,C_{t} \,E$$

**Figure 17:**
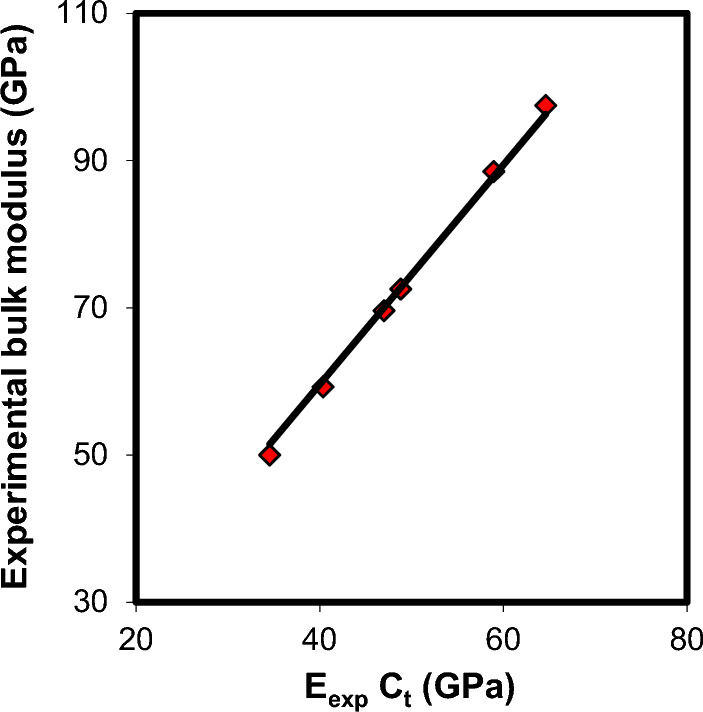
Relation between experimental bulk modulus and quantity $\left( {C_{t\,} E_{\exp } } \right)$ in xCdO–(1−x)[0.5PbO + 0.5Bi_2_O_3_] glasses. The solid line represents the least-square fitting of the data.

In these equations, $m$ and $n$ are constants in Mie’s atomic potential-energy equation ($\varphi (r) = - \frac{a}{{r^{m} }} + \frac{b}{{r^{n} }}$,$a$ and $b$ are two constants and $r$ is the distance between cation and anion), $\beta = {{\left( {r_{A} + r_{O} } \right)^{3} } \mathord{\left/ {\vphantom {{\left( {r_{A} + r_{O} } \right)^{3} } {\left( {y\,r_{A}^{3} + z\,r_{O}^{3} } \right)}}} \right. \kern-0pt} {\left( {y\,r_{A}^{3} + z\,r_{O}^{3} } \right)}}$ is a factor for an oxide $A_{y} O_{z}$ with cation ionic radius $r_{A}$ and oxygen ionic radius $r_{O}$. It is important to demonstrate the applicability of Eq. (4) for the present glasses. Figure 17 shows how the experimental bulk modulus varies with the quantity $\left( {C_{t\,} E_{\exp } } \right)$. As can be seen, the $K_{\exp }$−$C_{t\,} E_{\exp }$ relationship reveals a linear proportionality. The fitted curve in the figure can be represented by the following semi-empirical formula:

5 $$K_{\exp } \, = 1.49\,\,C_{t\,} E_{\exp }$$

with R^2^ = 0.996 and $\left[ {(m - n)\,m/24\pi } \right]\,\beta \,\, = 1.49$. These results show that the correlation between bulk and Young’s moduli of xCdO–(1−x)[0.5PbO + 0.5Bi_2_O_3_] glasses can be achieved through the total theoretical packing density.

## Conclusions

Cadmium lead bismuthate glasses in the system xCdO–(1−x)[0.5PbO + 0.5Bi_2_O_3_] (40 mol% ≤ x ≤ 90 mol%) were successfully prepared and their properties are characterized using XRD, FTIR and ultrasonic pulse-echo techniques. The inclusion of CdO has linearly decreased the ultrasonic velocity, elastic moduli, micro-hardness and Debye temperature increase show a linear increase. FTIR analysis clearly suggested that CdO plays a network modifier role, whereas Bi_2_O_3_ and PbO both play the role of network formers. The presence of BiO_3_ and PbO_4_ units is declined in favor of the presence of BiO_6_ units as CdO content increases. This modified the structure and increased the cross-link density and rigidity of the glass. Finally, a good correlation was achieved between elastic moduli and the most significant compositional parameters, like molar volume, fractal bond connectivity, packing density and dissociation energy per unit volume.

## Data Availability

No data associated in the manuscript. All original data supporting figures and discussion are listed in the Tables, which are included in the manuscript.
